# Posterior reversible encephalopathy syndrome in children with malignancies – a single-center retrospective study

**DOI:** 10.3389/fneur.2023.1261075

**Published:** 2023-11-03

**Authors:** Aleksandra Kiermasz, Magdalena Zapała, Bartosz Zwiernik, Angelika Stręk-Cholewińska, Magdalena Machnikowska-Sokołowska, Agnieszka Mizia-Malarz

**Affiliations:** ^1^Department of Oncology, Hematology, and Chemotherapy, Upper Silesia Children’s Care Health Centre, Katowice, Poland; ^2^Students’ Research Group, Department of Pediatrics, Medical University of Silesia, Katowice, Poland; ^3^Department of Diagnostic Imaging, Radiology and Nuclear Medicine, Upper Silesia Children’s Healthcare Center, Medical University of Silesia, Katowice, Poland; ^4^Department of Pediatrics, Medical University of Silesia, Upper Silesia Children’s Care Health Centre, Katowice, Poland

**Keywords:** posterior reversible encephalopathy syndrome, malignancies, children, clinical symptoms, magnetic resonance

## Abstract

**Background:**

Posterior reversible encephalopathy syndrome (PRES) diagnosis relies on clinical and radiological characteristics. Clinical manifestations include focal neurologic deficits, hemiparesis, seizures with symptoms of intracranial hypertension, headache, nausea, vomiting, and visual field disturbances. The majority of patients have typical changes in magnetic resonance imaging. The epidemiology and outcomes of PRES in the pediatric cancer population have not been well described. Most of the available data are from retrospective analyses.

**Objective:**

The aim of our study was to evaluate the clinical and radiological presentation as well as the outcome of PRES in children treated for cancers in a single center.

**Methods:**

We analyzed data from 1,053 patients diagnosed with malignancies in a single center over 15 years to determine the incidence of PRES.

**Results:**

19/1053 (1.8%) patients developed PRES. The diagnosis was accompanied by a range of clinical symptoms including hypertension, seizures, altered mental status, and headaches. Magnetic resonance imaging was performed in all patients, and 14/19 (73.7%) exhibited typical findings consistent with PRES. Four patients (21.0%) required treatment in the Intensive Care Unit.

**Conclusion:**

1. Posterior reversible encephalopathy syndrome (PRES) is a rare but significant complication in children with cancer.
2. There is a clear need to establish clinical criteria for PRES to improve the diagnosis and treatment of patients with PRES, particularly in the pediatric oncological population.
3. Further studies are needed to identify the risk factors for recurrent PRES, particularly in pediatric cancer patients undergoing chemotherapy or immunosuppressive treatment.

## Introduction

Posterior reversible encephalopathy syndrome (PRES) is diagnosed based on the presence of clinical symptoms and specific radiological changes (1–3). Despite a limited understanding of PRES epidemiology, children with cancer or those who have undergone allogeneic hematopoietic stem cell transplantation (HSCT) appear to be at increased risk for developing PRES. However, few studies have determined the incidence of PRES in pediatric populations, with estimates ranging around 0.04% (2). Among the various risk factors associated with PRES, elevated blood pressure is the most commonly described (3). Other risk factors include autoimmune diseases, renal diseases, anemia, and organ or hematopoietic stem cell transplantation (HSCT) (1–5). It was also reported in patients receiving immunospuppressive and cytotoxic treatments such as cyclosporin, cisplatin, cytarabine, high-dose methotrexate, gemcytabine, FK506, interferon α, erythropoietin, tacrolimus ans L-asparaginase (6). Several potential pathophysiological explanations for PRES have been proposed, with endothelial dysfunction and the theory of hypertension-induced breakdown in cerebral autoregulation being the most accepted mechanisms (2–4). Diagnosis of PRES relies on clinical and radiological characteristics, with neurological manifestations including focal neurologic deficits, hemiparesis, seizures with symptoms of intracranial hypertension, headache, nausea, vomiting, and visual field disturbances (3). Magnetic resonance imaging (MRI) is the standard imaging modality, typically showing involvement of the parieto-occipital region, although other areas such as the temporal or frontal lobes and posterior fossa may also be affected (5, 7). The term “reversible” stems from the fact that neurological and neuroimaging findings often spontaneously improve with the initiation of treatment, which typically involves antihypertensive and anti-edema measures (5). Unfortunately, data suggest that there is a subset of patients with PRES who may experience an unfavorable course with incomplete recovery, leading to permanent neurological deficits and even death (3, 8).

## Subjects and methods

This retrospective observational study included a total of 1,053 patients, ranging in age from 1 month to 17.8 years, who were diagnosed and treated for malignancy between June 2008 and December 2022 at the Oncology, Hematology, and Chemotherapy Department of the Upper Silesia Children’s Healthcare Center. Patients who presented with symptoms suggestive of PRES (hypertension and/or encephalopathy) underwent screening with magnetic resonance imaging (MRI). The diagnosis of PRES was established based on clinical symptoms supported by radiographic evidence. A subgroup of 19 children (1.8%) with PRES syndrome was identified, and additional data were collected from their medical records. The collected data were analyzed to determine the incidence in PRES, which is presented as medians and ranges or as frequencies.

## Results

The study group consisted of 19 patients diagnosed with PRES; age 2.5–11 years old (median 6.0); gender F/M 8/11. Among the studied group, both solid tumors and hematological malignancies were diagnosed, with acute lymphoblastic leukemia being the most common cancer associated with PRES (*n* = 9) (Table 1). Gender, age, and multiple diseases were assessed for their association with PRES, but none of these factors were found to be statistically significant (*p* > 0.05).

**Table 1:** Types and localization of primary diagnosis in all 19 patients with PRES.

Diagnosis	PRES Group (*N* = 19)
Acute Lymphoblastic Leukemia (ALL)	9 (47.4%)
Non Hodgkin Lymphoma (NHL)	2 (10.5%)
Acute Myeloid Leukemia	1 (5.3%)
Brain Tumors	4 (21.1%)
Neuroblastoma	1 (5.3%)
Nephroblastoma	1 (5.3%)
PNET	1 (5.3%)

Localization of Primary Disease	PRES Group (*n* = 19)
Bone Marrow	9 (47.3%)
Mediastinum	1 (5.3%)
Central Nervous System	4 (21.1%)
Retroperitoneal Space	2 (10.5%)
Peritoneal Cavity	1 (5.3%)
Lymph nodes	1 (5.3%)
Nasopharynx tumor/Bone Marrow	1 (5.3%)

The median time from the onset of malignancy treatment to the diagnosis of PRES was 81 days, with an average of 226 days. A comparison of this median between groups with different primary diagnoses revealed a significantly shorter time to develop PRES in the hematological group compared to the brain tumor group (81 days vs. 396.5 days). The receipt of intrathecal chemotherapy was not found to be associated with PRES (*p* > 0.05). All patients received standard protocol treatment based on their oncological diagnosis. Prior to the episode of PRES, 5 children (26.3%) received supportive radiotherapy, and 2 patients (10.5%) underwent hematopoietic stem cell transplantation (HSCT). Children with acute lymphoblastic leukemia (ALL), non-Hodgkin lymphoma, and relapsed ALL had all received corticosteroids at the time of PRES onset.

### Clinical symptoms

The analysis also focused on the type and frequency of clinical complaints to better characterize the prodromal symptoms and course of the illness (Table 2). Among the patients, the most common symptoms were episodes of hypertension and disturbances of consciousness, which were observed in 15 out of 19 cases (78.9%). Apathy was the next most common symptom, occurring in 42.1% of children (8 out of 19), followed by visual changes, headache, and nausea or vomiting. The median systolic pressure was 145 (110–180) and the diastolic pressure was 100 (70–120).

**Table 2:** Number of symptoms experienced by patients with PRES.

Symptoms	Patients from PRES Group (*N* = 19)
Hypertension	15 (78.9%)
Mental Disorder	15 (78.9%)
Seizures	15 (78.9%)
Epileptic State	6 (31.6%)
Apathy	8 (42.1%)
Visual changes	6 (31.6%)
Headache	3 (15.8%)
Vomiting	3 (15.8%)
Nausea	2 (10.5%)

### MRI results

**Figure 1:**
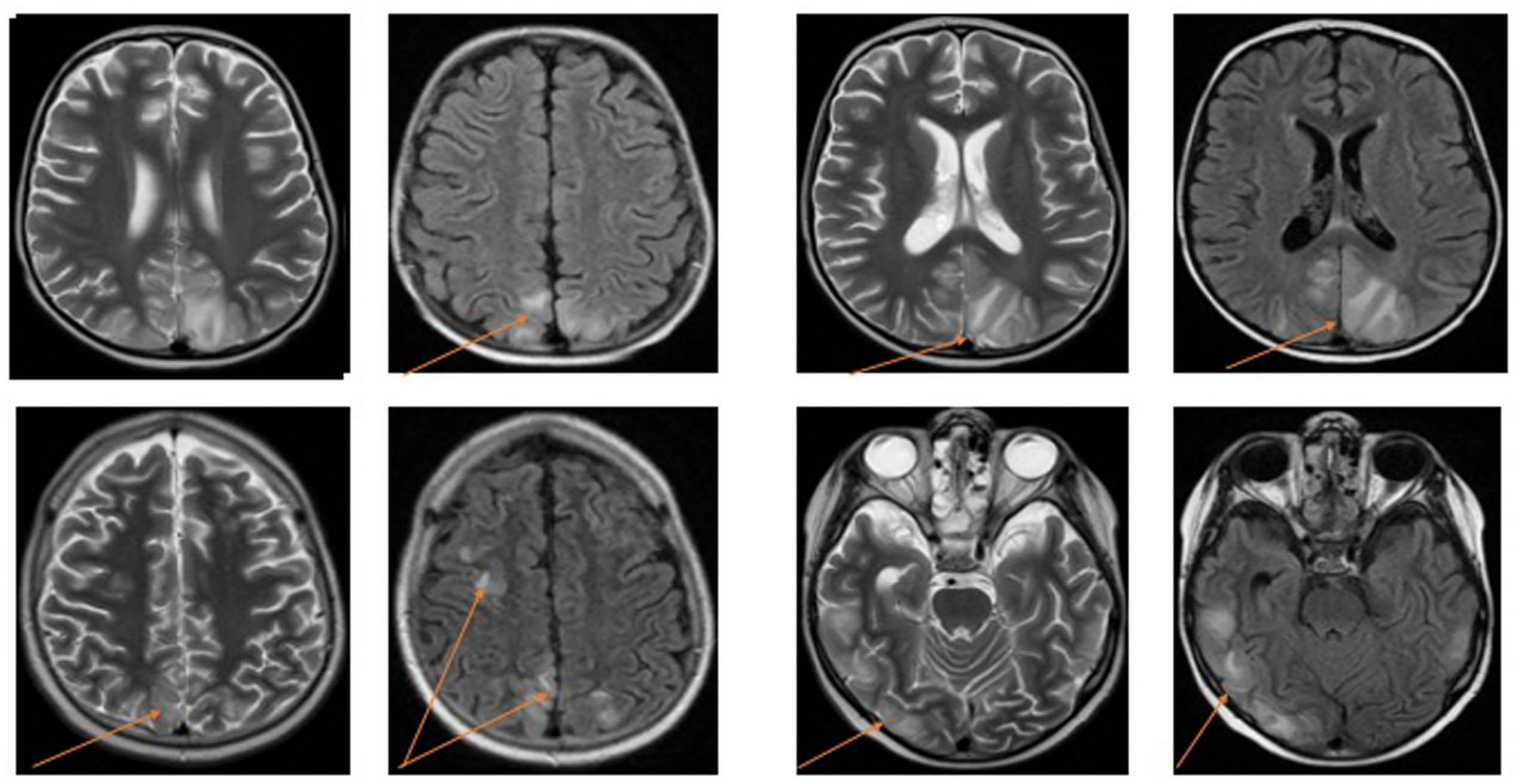
PRES changes in the brain, MRI images from 2 patients.

The majority of findings in the group were consistent with typical PRES characteristics, including subcortical areas of high signal intensity on fluid-attenuated inversion recovery (FLAIR) and T2-weighted images (Table 3). The most frequently affected regions were the occipital, parietal, and frontal lobes. Among the observed findings, 63.20% were symmetrical and bilateral. Additionally, four patients exhibited diffuse lesions in both hemispheres of the cerebellum (Figure 1).

**Table 3:** Number of MRI findings associated with patients with PRES.

MRI findings	Patients from PRES Group (*N* = 19)
Edema	6 (31.6%)
Frontal lobe	11 (57.9%)
Temporal lobe	4 (21.1%)
Parietal lobe	13 (68.4%)
Occipital lobe	14 (73.7%)
Cerebellum	4 (21.1%)
Bilateral	12 (63.2%)

### Laboratory findings

The median blood cell counts revealed the presence of anemia and thrombocytopenia, which are consistent with the underlying diseases. Additionally, 6 patients (31.6%) were found to be neutropenic at the time of PRES onset. Elevated levels of C-reactive protein (CRP) and aminotransferases were retrospectively reported in 9 out of 19 cases (47.4%) and 4 out of 19 cases (21.1%), respectively. Hyponatremia was the most frequently observed electrolyte disorder, reported in 36.8% of cases. Hypomagnesemia and hyperkalemia were each detected in 15.8% of children. Hypernatremia and hypophosphatemia were reported in 5.3% of cases (Table 4). We did not perform lumbar puncture in our patients.

**Table 4:** Value of blood pressures and laboratory evaluation in first day of PRES diagnosis.

Examination	Value
Blood pressures (mmHg)	
Systolic Pressure	145 (110–180)
Diastolic Pressure	100 (70–120)
Lab Values	
Hemoglobin (g/dL)	10.80 (7.2–19.3)
WBC (×10^3^/μL)	2.98 (0.1–14.57)
PLT (×10^3^/μL)	75.5 (7.8–273.0)
Electrolytes	
Hyponatremia Yes/No	7/12 (36.8%)
Hypokalemia Yes/No	2/17 (10.5%)
Hypomagnesemia Yes/No	3/16 (15.8%)
Biochemistry	
↑CRP Yes/No	9/10 (47.4%)
↑AlAT Yes/No	4/15 (21.1%)
↑AspAT Yes/No	4/15 (21.1%)

Seizure control and the restoration of a normal level of consciousness were achieved in all the children studied within 1–2 weeks. One case of recurrence was reported, occurring 9 days after the initial attack. This patient had been diagnosed with acute lymphoblastic leukemia (ALL), and PRES developed during induction therapy.

Antihypertensive medications were administered to control hypertension in all patients. Additionally, 12 out of 19 patients (63.2%) received anti-edema treatment in the form of 20% mannitol infusions. Clonazepam was the most commonly used medication for seizure prevention. Four out of 19 children (21.2%) required transfer to the Intensive Care Unit. Among the patients, only four needed ongoing chemotherapy, which was temporarily suspended for a maximum of 2 weeks after the diagnosis of PRES.

Unfortunately, one patient diagnosed with PRES passed away during hospitalization on intensive care unit due to increasing cerebral edema caused by PRES and clostridium sepsis.

## Discussion

Posterior reversible encephalopathy syndrome (PRES) is a relatively rare neurological and radiological complication in the pediatric population, and its etiology encompasses a wide range of possibilities (1–6). Our study examined 19 children diagnosed with PRES over 15 years. The most common underlying diseases in our group were hematological malignancies, with acute lymphoblastic leukemia (ALL) being the most frequently observed. All the patients with acute lymphoblastic leukemia (ALL), non-Hodgkin lymphoma, and relapsed ALL had all received corticosteroids at the time of PRES onset. The incidence of PRES in our analysis was 1.8%, which translates to 18.5 cases per 1,000 patients per year. A comparison of the groups with different primary diagnoses revealed a significantly shorter time to develop PRES in the hematological group compared to the brain tumor group. All the studied patients experienced clinical recovery, with only one case of recurrence reported. We were able to characterize the clinical symptoms and MRI findings in our group.

Previous studies have retrospectively investigated the incidence rate of PRES in children, with estimates ranging from 0.4% in pediatric intensive care unit admissions to 0.7% in pediatric patients with cancer and 0.04% in the pediatric population in the USA (2, 9, 10). While the incidence in our study (1,8%) may appear high, it is important to consider the specific nature of our group, which consisted of a single-center pediatric population. Furthermore, the lack of standardized diagnostic criteria for PRES, given its diverse manifestations (both clinical and radiological) among patients, adds to the difficulty of determining the true incidence. Thus, it is crucial to understand the presenting signs, symptoms, and etiological factors (such as associated treatments and underlying diseases) that may predispose a child to PRES.

In our analysis of clinical presentation and radiological findings, we observed the most frequently reported changes that were consistent with the existing literature on PRES (1–3, 11, 12). MRI was the confirmation method used in all patients, and the typical involvement of the parietal, occipital, and frontal lobes was commonly observed (13). Four patients also exhibited cerebellar involvement, which, although less common, has been reported in cases of PRES (7, 13). The top three symptoms in our study were altered mental status, convulsions, and hypertension. Notably, these symptoms were often observed in combination, suggesting complex manifestations of PRES with associated neurological deficits. This underscores the importance of considering PRES in the differential diagnosis of neurological deficits. Hypertension is the most commonly observed manifestation, increased blood pressure may disrupt cerebral autoregulation, which is one of the proposed mechanisms in PRES pathophysiology (14). In literature, PRES has also been reported in cases with normal or marginally elevated blood pressure. In such cases, cancer, chemotherapy, or immunosuppressive treatments may contribute to PRES through their damaging effects on the vascular endothelium (15). In our study the majority of patients manifested hypertension. All the patients in our study had a history of immunomodulatory treatment or chemotherapy, and the majority of children with ALL developed PRES during the induction phase of chemotherapy. Previous studies have also suggested induction chemotherapy as a significant risk factor for PRES in children with leukemia (16–18). However, there have been reports of PRES occurring during the consolidation phase as well (19). Several chemotherapeutic drugs, including vincristine, L-asparaginase, cyclophosphamide, high-dose methotrexate, and cytarabine, have been associated with PRES, but none have been identified as the definitive cause in children with leukemia (16–18). In our group 63% of patients with hematological malignancies (7 of 11 patients) received L-Asparaginase 3–14 days, 81% (9 of 11 patients) received intrathecal methotrexat and all received corticosteroids before the episode of PRES. The combination of drug toxicities is likely to contribute to the development of PRES. Unfortunately, modifying drug doses or discontinuing therapy entirely may not always be feasible, particularly when combating cancer. The pathogenesis of this syndrome remains incompletely understood, highlighting the need for further research. In our study, we observed only one case of a second episode of PRES in patients with ALL (16, 20–22). There is still uncertainty as to whether the continued administration of drugs after the initial episode of PRES contributes to recurrence (18, 19).

Most PRES cases demonstrate reversible and favorable outcomes (8, 14, 23–26). However, the mortality rate varies widely depending on the population studied, ranging from 3.2% in children to as high as 16% in adults (2, 5). Only one patient died in our study group which is 5.2%. It is higher than in the literature, possibly because of the small number of patients in our study group. Certain risk factors, such as autoimmune conditions, hypertensive crisis, renal failure, multi-organ failure, and infection, may indicate a worse prognosis or irreversible PRES (14, 23, 25).

This study has some limitations, as it is retrospective and conducted in a single center over 15 years, focusing specifically on pediatric malignancies. Nonetheless, it provides important insights into the diagnosis and outcomes of PRES in this population. Future studies should aim to include a larger number of children from multiple centers to further advance our understanding of PRES.

## Conclusion

1. Posterior reversible encephalopathy syndrome (PRES) is a rare but significant complication in children with cancer.
2. Children suffering from acute lymphoblastic leukemia in the course of induction treatment are the most vulnerable group of pediatric oncological patients for PRES.
3. There is a clear need to establish clinical criteria for the improved diagnosis and treatment of patients with PRES, particularly in the pediatric oncological population.
4. Further studies are needed to identify the risk factors for recurrent PRES, particularly in pediatric cancer patients undergoing chemotherapy or immunosuppressive treatment.

## Data availability statement

The original contributions presented in the study are included in the article/supplementary material, further inquiries can be directed to the corresponding author.

## Ethics statement

Ethical approval was not required for the study involving human samples in accordance with the local legislation and institutional requirements. Written informed consent for participation in this study was provided by the participants’ legal guardians/next of kin.

## Author contributions

AM-M: Conceptualization, Formal analysis, Supervision, Writing – original draft, Writing – review & editing. AK: Resources, Writing – original draft. MZ: Formal analysis, Project administration, Writing – original draft. BZ: Formal analysis, Resources, Writing – original draft. AS-C: Formal analysis, Resources, Writing – review & editing. MM-S: Resources, Writing – review & editing.
